# Supplementary figures and images for: Combining GAN with reverse correlation to construct personalized facial expressions
Source: PLoS One. 2023 Aug 25;18(8):e0290612. doi: 10.1371/journal.pone.0290612 (PMC10456187; doi:10.1371/journal.pone.0290612)

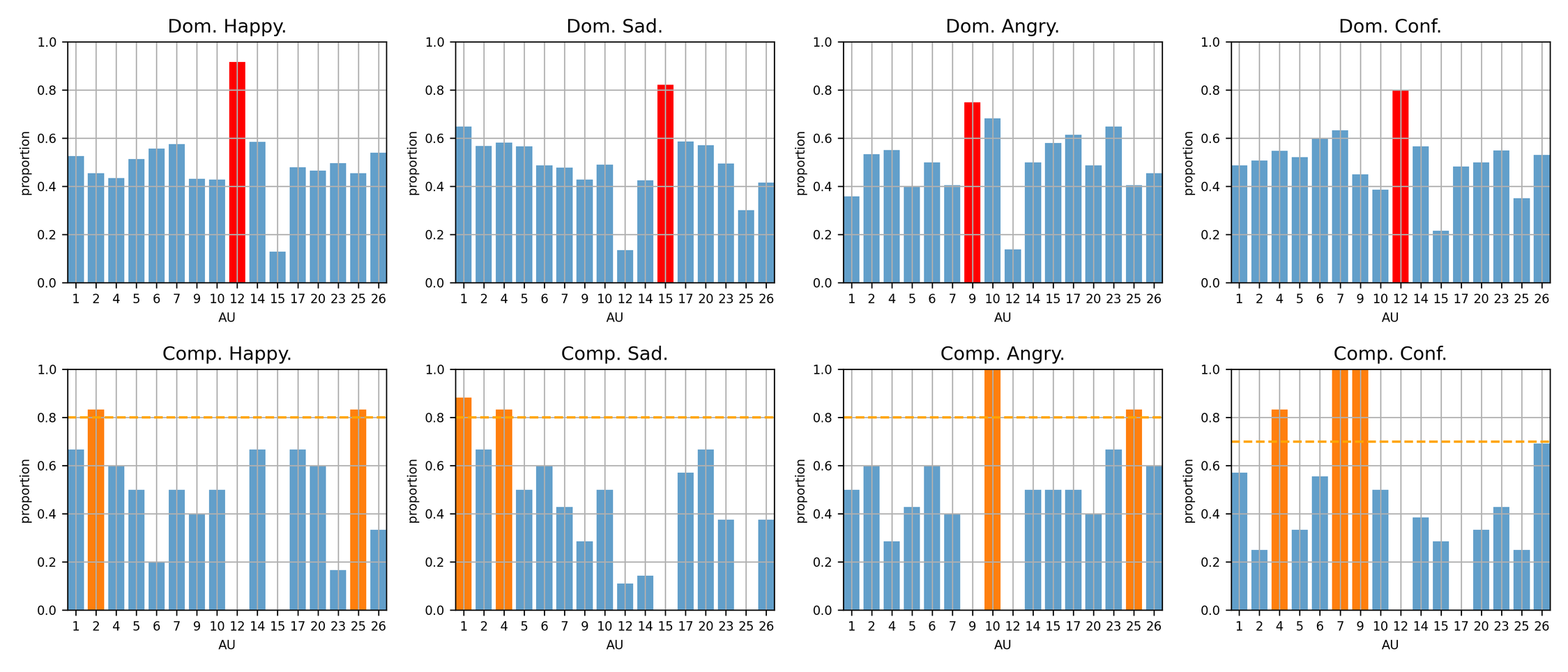

Supplement: S1 Fig — (TIF) [file pone.0290612.s001.tif]

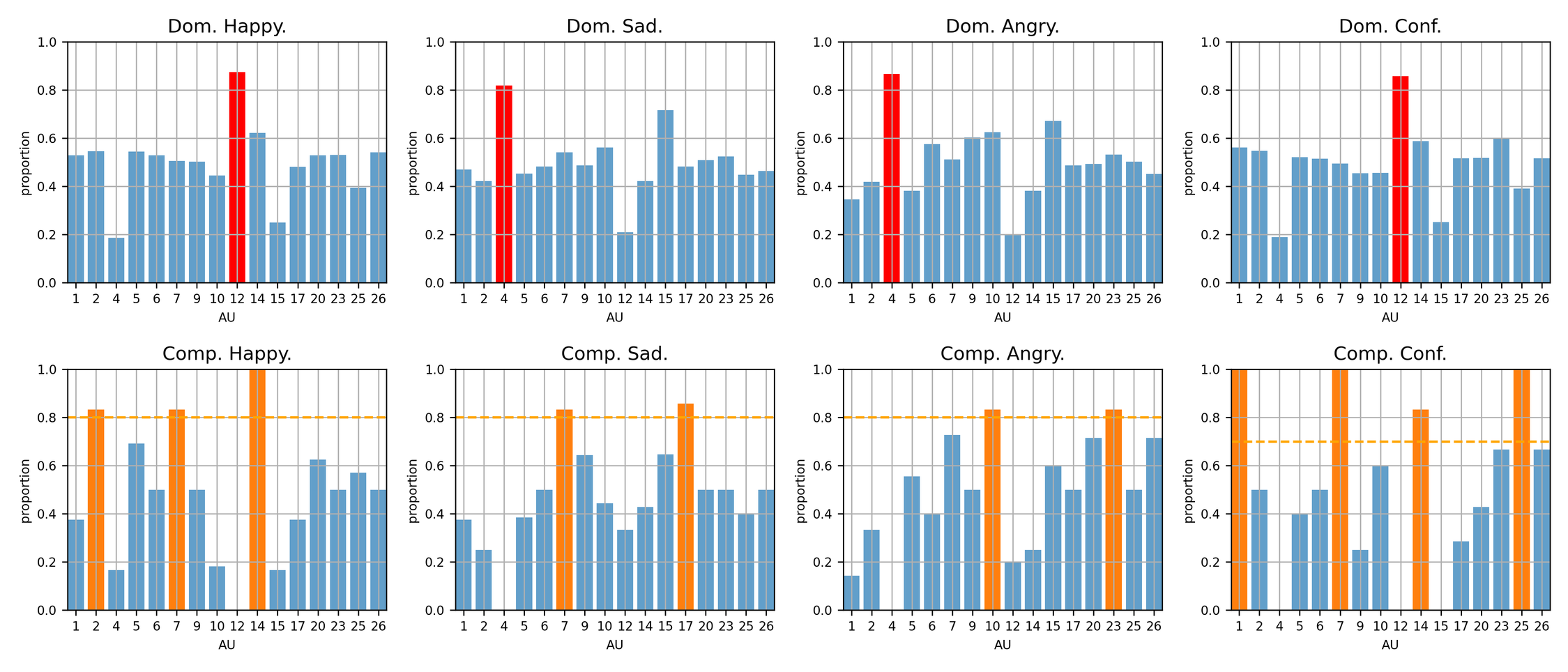

Supplement: S2 Fig — (TIF) [file pone.0290612.s002.tif]

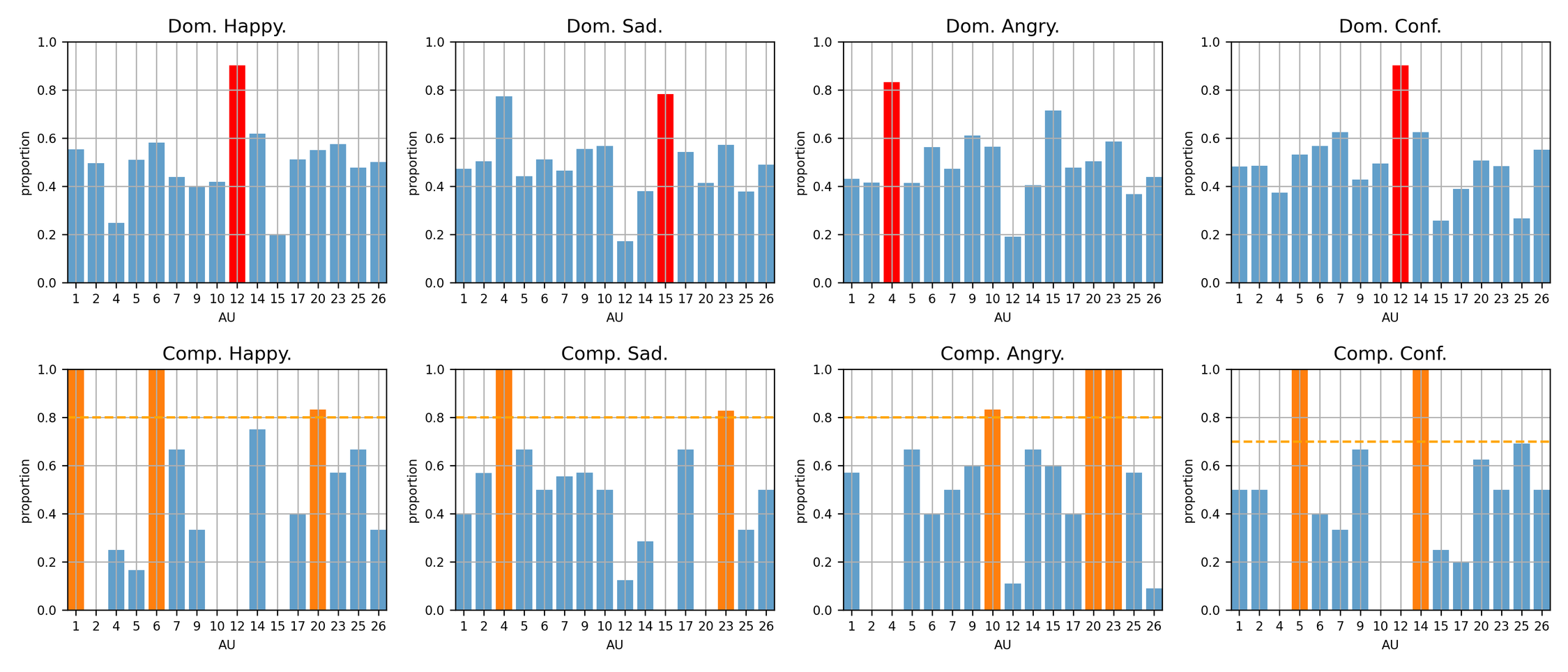

Supplement: S3 Fig — (TIF) [file pone.0290612.s003.tif]

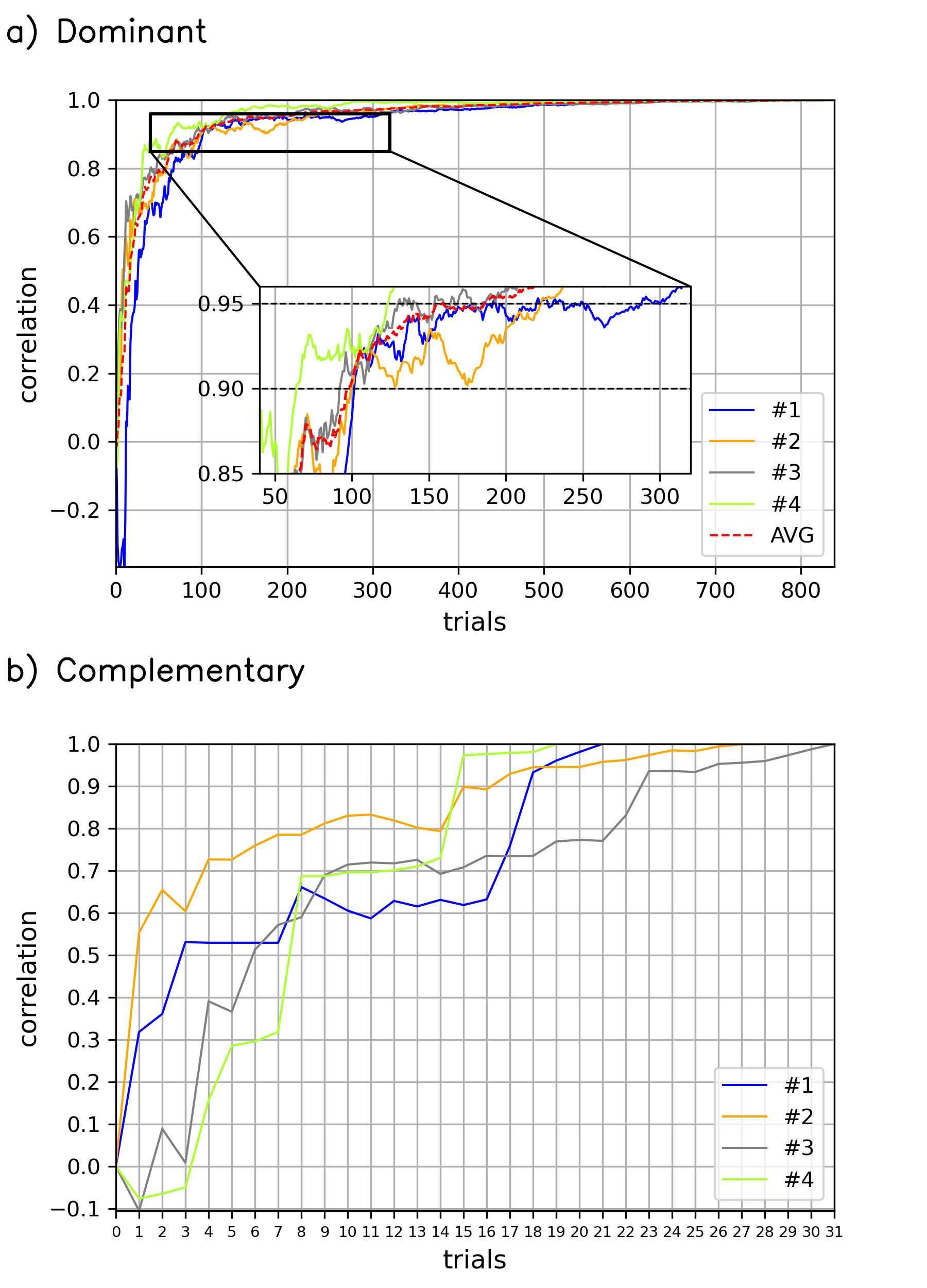

Supplement: S4 Fig — (TIF) [file pone.0290612.s004.tif]

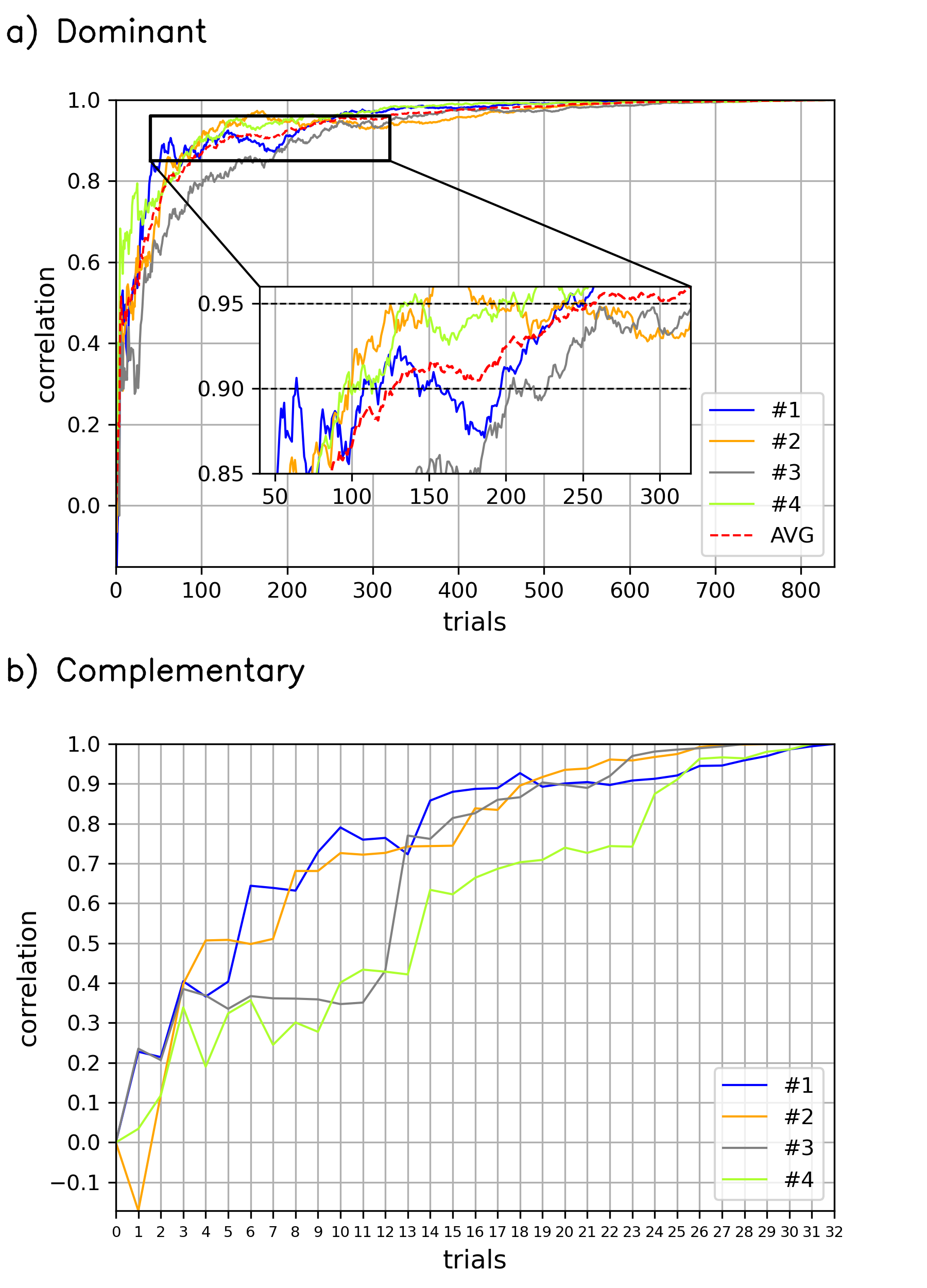

Supplement: S5 Fig — (TIF) [file pone.0290612.s005.tif]

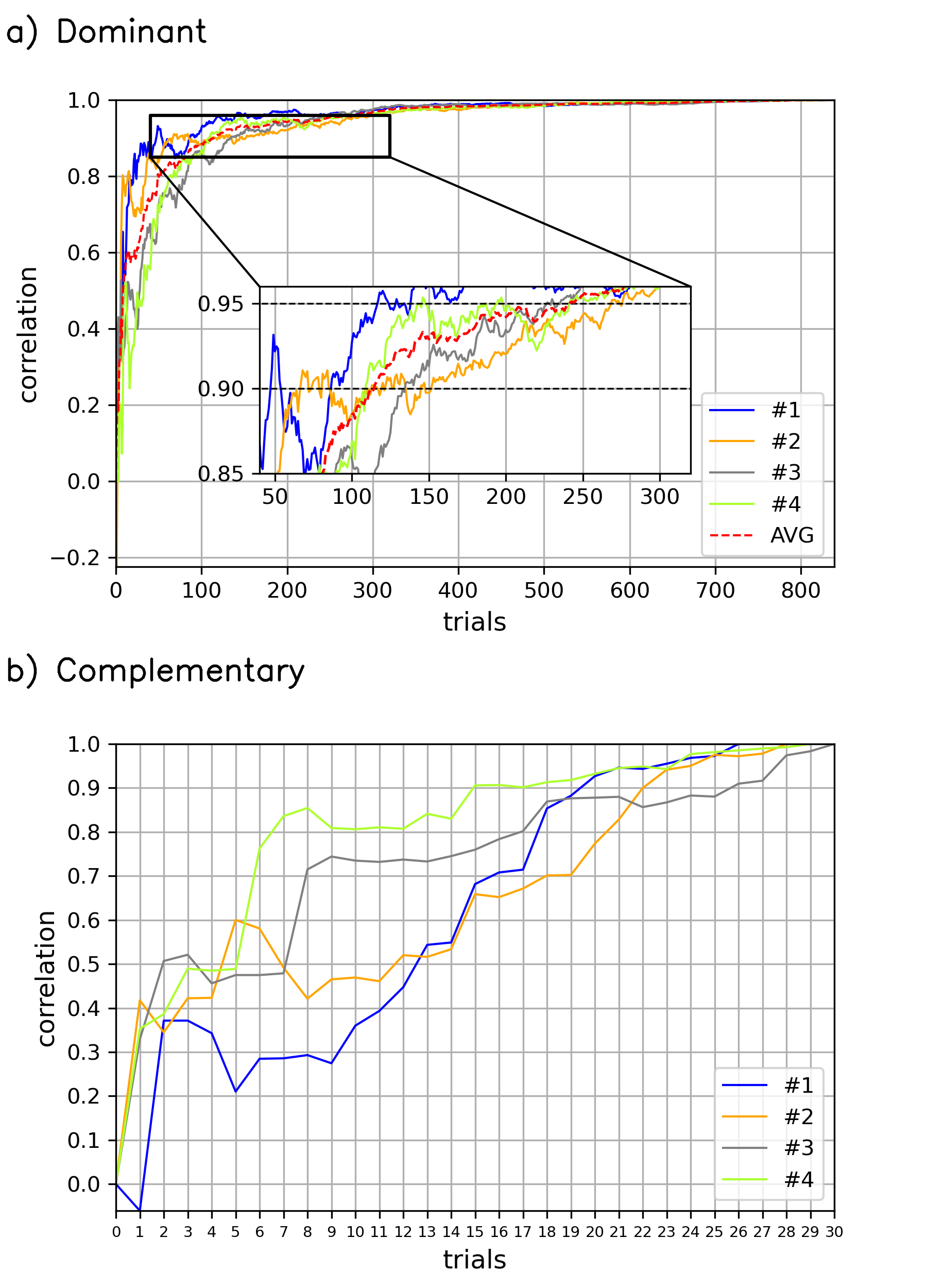

Supplement: S6 Fig — (TIF) [file pone.0290612.s006.tif]
