# Supplementary material for: Combining GAN with reverse correlation to construct personalized facial expressions
Source: PLoS One. 2023 Aug 25;18(8):e0290612. doi: 10.1371/journal.pone.0290612 (PMC10456187; doi:10.1371/journal.pone.0290612)
Supplement: S1 Appendix — (PDF) [file pone.0290612.s007.pdf]

Supporting information: GANimation

Differing from manipulating a discrete number of facial expressions (global attributes), GANimation [1] (in Fig 1) is based on Action Units (AU) [2], which describe a facial expression by anatomical facial movements.

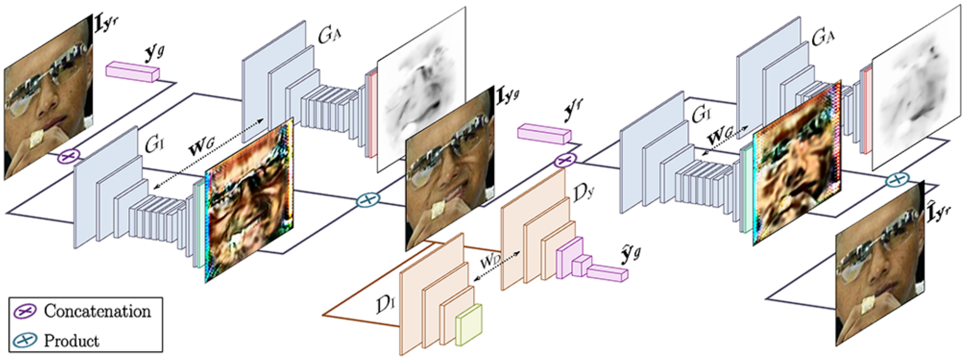

Fig 1. Overview of GANimation copied from the literature [1]. This model consists of three main blocks: a generator G to regress attention (G_A) and color masks (G_I), and a critic D_I to evaluate the quality of the generated image I_{y_g} and its photo-realism; and an expression estimator D_y to penalize differences between the desired conditioning expression y_g and its fulfillment $\hat{y}_g$. Note that I_{y_r} is an input RGB image, and I_{y_g} is the output. The generator G is applied twice, first to map the input image I_{y_r} to the output image I_{y_g}, and then to render it back from the output I_{y_g} to the reconstructed image $\hat{I}_{y_r}$. y_g represents the vector of AUs that should be activated for the output image I_{y_g}. y_r is the AUs of the input image I_{y_r}.

In detail, this model can edit 16 different AUs (6 upper face AUs and 10 lower face AUs) from Facial Action Coding System [2]: AU1 Inner Brow Raiser, AU2 Outer Brow Raiser, AU4 Brow Lowerer, AU5 Upper Lid Raiser, AU5 Cheek Raiser, AU6 Cheek Raiser, AU7 Lid Tightener, AU9 Nose Wrinkler, AU10 Upper Lip Raiser, AU12 Lip Corner Puller, AU14 Dimpler, AU15 Lip Corner Depressor, AU17 Chin Raiser, AU20 Lip stretcher, AU23 Lip Tightener, AU25 Lips part, and AU26 Jaw Drop.

References

1. Pumarola A, Agudo A, Martinez AM, Sanfeliu A, Moreno-Noguer F. GANimation: Anatomically-aware Facial Animation from a Single Image. In: Proceedings of the European Conference on Computer Vision (ECCV); 2018.

2. Friesen E, Ekman P. Facial action coding system: a technique for the measurement of facial movement. Palo Alto. 1978;3.
