# Supplementary material for: Combining GAN with reverse correlation to construct personalized facial expressions
Source: PLoS One. 2023 Aug 25;18(8):e0290612. doi: 10.1371/journal.pone.0290612 (PMC10456187; doi:10.1371/journal.pone.0290612)
Supplement: S2 Appendix — (PDF) [file pone.0290612.s008.pdf]

Supporting information: Schulze method

Schulze method is a new single-winner election method for internal elections and referendums. This method has been adopted by the Pirate Party of Sweden, the Wikimedia Foundation, the Debian project, the "Software in the Public Interest" project, the Gentoo project, and many other private organizations. This method can be divided into 3 steps: voting, pairwise preferences, and strongest paths. We explain this method according to our "subjective evaluation experiment: ranking".

Voting. The voting can be a ranking task like our subjective evaluation experiment. For each emotion (happiness, sadness, anger, and self-confidence), the participants rank each of the listed prototypes (personalized prototypes of the observers and state-of-the-art prototypes if they exist).

Pairwise preferences of Schulze method. For each emotion, based on the ranking results, we count the pairwise preferences between each of the prototypes. For example, for happiness, when comparing the prototypes of observer #1 and observer #2, there are 16% of the participants who prefer the prototype of observer #1 to the prototype of observer #2, and there are 84% of the participants who prefer the prototype of observer #2 to the prototype of observer #1. So the matrix of the pairwise preferences can be created (Fig 1).

We can notice the **cyclic preferences** for sadness. 51% of the participants prefer the prototype of observer #2 to the prototype of observer #4, 57% of the participants prefer the prototype of observer #4 to the state-of-the-art prototype of Yu [1], whereas 52% of the participants prefer the state-of-the-art prototype of Yu [1] to the prototype of observer #2. Thus we can not identify the preferences between these three prototypes. However, by calculating the strongest paths, the Schulze method can solve this problem and obtain the final ranking with a single winner.

Strongest paths. The directed graph Fig 1 shows that there can be multiple paths to go from one node (i.e., one prototype in our case) to another node (i.e., another prototype in our case). The arrow indicates that each path has only one direction. For example, in the directed graph of anger (Fig 1(c)), the path from the prototype of observer #1 to the prototype of observer #4 can be multiple:

1. from "#1" to "#2" to "#4";
2. from "#1" to "#2" to "#3" to "#4";
3. from "#1" to "#3" to "#4";
4. from "#1" to "#4";
5. from "#1" to "Yu" to "#4".

We define the strength of the path as the minimum preference in the path. The strengths of the above 5 possible paths are:

1. $min(70\%, 72\%) = 70\%$
2. $min(70\%, 78\%, 52\%) = 52\%$
3. $min(76\%, 52\%) = 52\%$
4. $min(78\%) = 78\%$

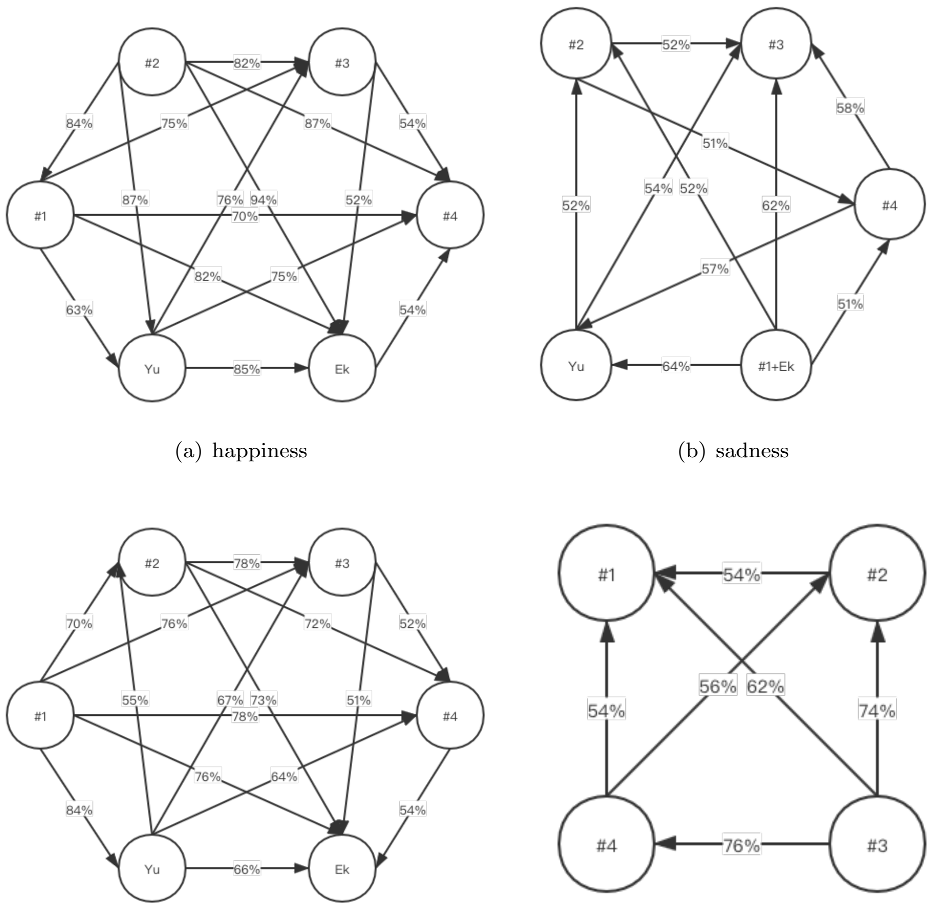

(a) happiness (b) sadness

(c) anger (d) confidence

Fig 1. Directed graph labeled with pairwise preferences. We use a directed line (arrow) to connect each pair of prototypes from the "preferred" prototype to the "less preferred" prototype. For example, for happiness, when comparing the prototypes of observer #1 and observer #2, there are 84% of the participants who prefer the prototype of observer #2 to the prototype of observer #1. So the directed line starts from observer #2 and ends at the prototype of observer #1. Similarly, based on this graph, we can infer that 16% (100% − 84%) of participants prefer the prototype of observer #1 to the prototype of observer #2. Note that for sadness, the prototype of observer #1 and the state-of-the-art prototype of Ekman are identical. Thus we merge them (denoted by "#1-Ek.").

5. $min(84\%, 64\%) = 64\%$

Thus in this example, the strongest path is the path with the maximum strength, i.e., the 4th path (from "#1" to "#4") with the strength 78%.

Finally, we can obtain the final preferences (i.e., the strength of the strongest path), as shown in Table 3 in the **main text**.

References

1. Yu H, Garrod OG, Schyns PG. Perception-driven facial expression synthesis. Computers & Graphics. 2012;36(3):152–162.
