# Supplementary material for: Combining GAN with reverse correlation to construct personalized facial expressions
Source: PLoS One. 2023 Aug 25;18(8):e0290612. doi: 10.1371/journal.pone.0290612 (PMC10456187; doi:10.1371/journal.pone.0290612)
Supplement: Attachment — Submitted filename: response to reviewers.pdf [file pone.0290612.s009.pdf]

To all reviewers and editors:

First, we would like to send my sincerest thanks to the reviewers and the editor for their efforts.

We followed the comments of the reviewers, majorly modified the related work and the methodology and reorganized the order of the subsection “convergency efficiency”. All the supporting information were updated accordingly.

In addition, a new subjective evaluation has been added and merged with the previous subjective evaluation in section 4.2.

I copied the reviews from PLOS ONE and answered each of them accordingly.

1. Please ensure that your manuscript meets PLOS ONE's style requirements, including those for file naming. The PLOS ONE style templates can be found at

https://journals.plos.org/plosone/s/file?id=wjVg/PLOSOne_formatting_sample_main_body.pdf and

https://journals.plos.org/plosone/s/file?id=ba62/PLOSOne_formatting_sample_title_authors_affiliations.pdf

We followed the guideline and edited the format.

2. Please note that PLOS ONE has specific guidelines on code sharing for submissions in which author-generated code underpins the findings in the manuscript. In these cases, all author-generated code must be made available without restrictions upon publication of the work. Please review our guidelines at https://journals.plos.org/plosone/s/materials-and-software-sharing#loc-sharing-code and ensure that your code is shared in a way that follows best practice and facilitates reproducibility and reuse.

The code will be available at https://yansen0508.github.io/emotional-prototype/.

3. Thank you for stating the following financial disclosure:

“Our work was funded by Randstad group (https://www.randstad.fr/, to Sen Yan, Catherine Soladié, and Renaud Seguier), Agence Nationale de la Recherche (ANR) REFLETS (https://anr.fr/, to Catherine Soladié, Jean-Julien Aucouturier, and Renaud Seguier), and European Research Council (ERC) PoC ACTIVATE 875212 and Fondation Pour l’Audition FPA RD-2018-2 (https://erc.europa.eu/homepage, to Jean-Julien Aucouturier).”

Please state what role the funders took in the study. If the funders had no role, please state: "The funders had no role in study design, data collection and analysis, decision to publish, or preparation of the manuscript."

If this statement is not correct you must amend it as needed.

Please include this amended Role of Funder statement in your cover letter; we will change the online submission form on your behalf.

The following state “The funders had no role in study design, data collection and analysis, decision to publish, or preparation of the manuscript.” has been added in the cover letter.

4. Thank you for stating the following in the Acknowledgments Section of your manuscript:

“Our work was funded by Randstad group (to S.Y., C.S., and R.S.), Agence Nationale de 524

la Recherche (ANR) REFLETS (to C.S., JJ.A., and R.S.), and European Research 525

Council (ERC) PoC ACTIVATE 875212 and Fondation Pour l’Audition FPA RD-2018-2 526

(to JJ.A.).”

We note that you have provided funding information that is currently declared in your Funding Statement. However, funding information should not appear in the Acknowledgments section or other areas of your manuscript. We will only publish funding information present in the Funding Statement section of the online submission form.

Please remove any funding-related text from the manuscript and let us know how you would like to update your Funding Statement. Currently, your Funding Statement reads as follows:

“Our work was funded by Randstad group (https://www.randstad.fr/, to Sen Yan, Catherine Soladié, and Renaud Seguier), Agence Nationale de la Recherche (ANR) REFLETS (https://anr.fr/, to Catherine Soladié, Jean-Julien Aucouturier, and Renaud Seguier), and European Research Council (ERC) PoC ACTIVATE 875212 and Fondation Pour l’Audition FPA RD-2018-2 (https://erc.europa.eu/, to Jean-Julien Aucouturier).”

Please include your amended statements within your cover letter; we will change the online submission form on your behalf.

Funding-related texts have been removed from the manuscript and added in the cover letter.

5. Please include your full ethics statement in the ‘Methods’ section of your manuscript file. In your statement, please include the full name of the IRB or ethics committee who approved or waived your study, as well as whether or not you obtained informed written or verbal consent. If consent was waived for your study, please include this information in your statement as well.

We add the ethics statement at the end of Method (the last paragraph of section 2.5).

6. Please upload a copy of Supporting Information Figure S1 fig and S2 fig which you refer to in your text on page 13.

We updated the supporting information.

S1-S3 Figs: Mental representation computation from the observer #1, #3, and #4 (previously S1 Fig).

S4-S6 Figs: Converging curves for happiness, sadness, and anger (previously S2 Fig).

In addition, we added S1 Appendix to introduce the details of GANimation and S2 Appendix to introduce the details of Schulze method.

Additional Editor Comments:

The manuscript presents innovative research on a facial expression generation method but requires significant revisions to improve clarity, comprehension, and validation. Key recommendations include: clarifying distinctions and research in attribute manipulation, improving image quality, expanding on current FEM techniques, explaining the GANimation model in more detail, conducting a comprehensive literature survey, simplifying methodological details and notation, addressing formatting and logical flow issues, relating results to chance level, providing clearer evidence for specific claims, improving figure resolution, explaining technical terms within the manuscript, and correcting minor errors. Addressing these concerns will significantly enhance the manuscript's quality and its potential for publication.

Thanks for these valuable comments. We followed these recommendations to majorly revise our manuscript.

[Note: HTML markup is below. Please do not edit.]

Reviewers' comments:

Reviewer's Responses to Questions

Comments to the Author

1. Is the manuscript technically sound, and do the data support the conclusions?

The manuscript must describe a technically sound piece of scientific research with data that supports the conclusions. Experiments must have been conducted rigorously, with appropriate controls, replication, and sample sizes. The conclusions must be drawn appropriately based on the data presented.

Reviewer #1: Partly

Reviewer #2: No

---

2. Has the statistical analysis been performed appropriately and rigorously?

Reviewer #1: No

Reviewer #2: No

---

3. Have the authors made all data underlying the findings in their manuscript fully available?

The PLOS Data policy requires authors to make all data underlying the findings described in their manuscript fully available without restriction, with rare exception (please refer to the Data Availability Statement in the manuscript PDF file). The data should be provided as part of the manuscript or its supporting information, or deposited to a public repository. For example, in addition to summary statistics, the data points behind means, medians and variance measures should be available. If there are restrictions on publicly sharing data—e.g. participant privacy or use of data from a third party—those must be specified.

Reviewer #1: No

Reviewer #2: No

---

4. Is the manuscript presented in an intelligible fashion and written in standard English?

PLOS ONE does not copyedit accepted manuscripts, so the language in submitted articles must be clear, correct, and unambiguous. Any typographical or grammatical errors should be corrected at revision, so please note any specific errors here.

Reviewer #1: Yes

Reviewer #2: Yes

---

5. Review Comments to the Author

Please use the space provided to explain your answers to the questions above. You may also include additional comments for the author, including concerns about dual publication, research ethics, or publication ethics. (Please upload your review as an attachment if it exceeds 20,000 characters)

Reviewer #1: This paper presents a facial expression generation method that utilizes a generative adversarial network model combined with reverse correlation to construct personalized facial expressions. This method has significant implications in the field of facial expression manipulation. While I believe the research is important, there are still some aspects that need to be modified and improved.

Thanks for the recognition of our work. We followed your suggestions to revise our manuscript.

-The Facial expression manipulation section would benefit from a clearer explanation of the differences between high-level attribute manipulation and low-level attribute manipulation, as well as a brief overview of existing research in these two areas.

The terms “high-level” and “low-level” seem to be confusing that causes this comment and the other comments from the reviewers. To eliminate the misunderstanding, we replace “high-level attributes” by “global attributes” and “low-level attributes” by “local attributes” and we add explanations of these terms. (See Section 1.3.1)

-The quality of the images provided in this paper could be improved by regenerating high-resolution images or using a vector image format to avoid distortion caused by scaling.

All the figures are regenerated to meet the author guideline. Please download the images via the links provided in the manuscript for better observation.

-While the weaknesses of FEM techniques are well-defined and specific, more details on current efforts to address these weaknesses would be helpful. For instance, what research or development is being done to improve fine control or personalize facial expressions?

We added all the proposed references by the reviewer and another 3 FEM papers in the scope of computer science into the subsection “Facial expression manipulation”. (See Section 1.3.1)

The proposed references by the reviewer: [1] GANs and Artificial Facial Expressions in Synthetic Portraits. [2] BMAnet: Boundary Mining With Adversarial Learning for Semi-Supervised 2D Myocardial Infarction Segmentation. [3] 3D Cartoon Face Generation with Controllable Expressions from a Single GAN Image. [4] Sparse to Dense Dynamic 3D Facial Expression Generation. [5] GCFSR: a Generative and Controllable Face Super Resolution Method Without Facial and GAN Priors.

Another 3 FEM papers: 1) “Dynamic facial expression generation on hilbert hypersphere with conditional Wasserstein generative adversarial net”, 2) “Person-specific joy expression synthesis with geometric method”, and 3) “Personalized expression synthesis using a hybrid geometric-machine learning method”.

-A detailed explanation and description of the GANimation model used in the Stimuli generation section, including its advantages and limitations, as well as the reasons for choosing this model, should be provided.

We added more explanations in the related works (Section 1.3.1, lines 150-169) and the supporting information (S1 Appendix).

In lines 150-156: the advantages and the reason for choosing GANimation.

In lines 157-169: the reason and the limitations.

-The authors should conduct a more thorough literature survey. Some relevant papers to consider include:[1] GANs and Artificial Facial Expressions in Synthetic Portraits. [2] BMAnet: Boundary Mining With Adversarial Learning for Semi-Supervised 2D Myocardial Infarction Segmentation. [3] 3D Cartoon Face Generation with Controllable Expressions from a Single GAN Image. [4] Sparse to Dense Dynamic 3D Facial Expression Generation. [5] GCFSR: a Generative and Controllable Face Super Resolution Method Without Facial and GAN Priors.

Thanks for providing these papers. [1,2,3,5] correspond to a more general concept rather than facial expression manipulation ([1]: the impact of StyleGAN in art [2]: medical image, [3]: (preprint paper) manipulating face image (not facial expression), [5]: super resolution on face image). [4] is relevant to facial expression manipulation.

We realize that the potential audiences may also confuse "facial expression" with "face", even we clarify "facial expressions" in the title and the entire introduction of our manuscript.

Thus, here are our proposals:

We add a new subsection "Preamble" (lines 90-98) in the related work and emphasize the differences between manipulating face and manipulating facial expression. The proposed papers [1,3,5] and the StyleGAN family (StyleGAN, StyleGAN2, StyleGAN3) are discussed in this subsection. [2] was added at the beginning of related work (line 85).

In addition, we add the proposed paper [4] and another 3 papers ("Dynamic facial expression generation on hilbert hypersphere with conditional Wasserstein generative adversarial net", "Person-specific joy expression synthesis with geometric method", and "Personalized expression synthesis using a hybrid geometric-machine learning method") into "global attribute manipulation" and "local attribute manipulation". (See Section 1.3.1)

Reviewer #2: Yan et al report an experiment in which face stimuli are generated by a generative adversarial network which can be fed with binary vectors that trigger the expression of so-called action units that lead to specific emotional expressions. The authors show such stimulus material to 4 participants and ask them to rate pairs of stimuli in terms of which of the pair seems a better expression of a given emotion. The authors then conduct a somewhat convoluted analysis to derive a "mental representation" of the emotions specific to each participant, test how much of their data is needed to obtain similar results and show the reconstructions to online participants (n = 217) in order to see to what extent these online participants agree with the reconstructions.
The manuscript is written in a quite confusing way, where for example the methodological details are spread out over multiple subsections that each explain a different sub-aspect of a problem, the figure captions are mostly unintelligible, and the authors choose very quirky analyses that they describe in a maximally convoluted way which I believe will reduce the intelligibility of what was done to pretty much any audience. None of the results are related to chance level, so it is hard to infer what the authors really have "found".

I however believe that if these issues are adressed, the manuscript could be improved considerably.

Thanks for the valuable comments about this paper. We responded to your concerns step by step and proposed our modifications.

Here are my concerns, in a loosely descending order of importance:

1) Logic of line 225 / "dominant action unit computation"
What is the rationale that motivated this? Why did the authors not simply consider a linear

model that predicts the decision as a function of the binary vector of AUs?
Is the mathematical set notation really necessary? It is extremely tough to read, and seems like it is greatly and unnecessarily overcomplicating relatively simple matters. Line 214 / 215 seems just a bit over the top. How exactly am I to understand the final mu[k] = i? Line 232: "knowing that for all AUj != AUd" -- I have no idea what is meant here.

- What is the rationale that motivated this?

  First of all, we clarified he key words in the section 1.1 “Requirements”, and majorly revised the related work (Section 1.3). All the following modifications in the methodology are based on the Sections 1.1 and 1.3.

  In Section 1.3.1, we explained the reason why we chose to edit the local attributes (e.g., AUs) rather than the global attributes (e.g., emotion label) to manipulate a facial expression.

  Thus, in terms of manipulating facial expression by editing AUs (i.e., local attributes in section 1.3.1), we proposed a two-step computation (first: dominant AU computation and second: complementary AUs computation). The purpose was added at the beginning of the section 2.3, (see line 247-257)

- Why did the authors not simply consider a linear model that predicts the decision as a function of the binary vector of AUs?

  The reason not considering a linear model is as follows:

  Although the pairs of stimuli will not be repeated for each experiment, individual stimulus is allowed to appear in different trials.

  For example in trial #1, there is a pair of stimuli {A,B}; in trial#2, the pair of stimuli is {A,C}. We can notice that “A” appears in two different trials. Depending on the subjective choice of the observer, this stimulus (A) can be “selected” and “not selected” from two different trials: e.g., A is selected in trial#1 but not in trial#2.

  So all the activated AUs within the stimulus A will also be annotated by “selected” and “not selected”.

  Then it will be difficult to classify which AU drives the observer’s perception (in a limited number of trials) by directly using a linear model, because the linear model can easily be confused by the opposite annotations on the same AU. Moreover, a linear model cannot describe the dependency between the AUs, but our approach can.

  Therefore, we propose the concept of dominant and complementary to compute step by step which AUs really affect the observer's decision. (See Section 2.3)

- Is the mathematical set notation really necessary? It is extremely tough to read, and seems like it is greatly and unnecessarily overcomplicating relatively simple matters.

  About math, our intention is that all text descriptions must be clear without ambiguity or misunderstanding. However, we did not expect to create difficulties in understanding.

  We modified and deleted the unnecessary formula. The previous Fig 2 is deleted.

- Line 214 / 215 seems just a bit over the top.

  Based on our revision, these notations no longer exist.

- How exactly am I to understand the final mu[k] = i?

  Based on our revision, this notation no longer exists.

- Line 232: "knowing that for all AUj != AUd" -- I have no idea what is meant here.

  We added more details about "complementary action units computation" (see lines 270-281).

  For the complementary AUs computation, we only focus on the non-dominant AU. The explanation of "knowing that for all AUj != AUd" can be found in lines 273 and 282-283.

It seems to me that the whole notation could be summarised in a few sentences like so: "For any given AU i, we considered the subset of trials in which only one stimulus of the pair had the AU i activated. We then divided the number of stimuli containing AU i that were chosen by the participant by the number of all trials in the subset. We defined the dominant AU as the one with the highest fraction.
We further considered trials in which both stimuli had a dominant AU activated. In a subset of these trials, a given additional AU j was active in only one of the two stimuli. We divided the number of stimuli that were chosen by the participant and had AU j active by the number of trials in this subset. For any AU j where this fraction exceeded .7 (self-confidence) or .8 (all other emotions), we counted AU j as a complementary AU."

Thanks for providing this summary. We majorly revised the method section.

We think that it will be easier to understand and eliminate ambiguity by using the equations to describe some long sentences proposed by the reviewer, such as "*we then divided the number of stimuli containing AU i that were chosen by the participant by the number of all trials in the subset.*" and "*We divided the number of stimuli that were chosen by the participant and had AU j active by the number of trials in this subset.*" Thus, we kept the Equations (1) to (4) and deleted the unnecessary notations.

If the authors wish to claim that the personalisation worked to some degree, then they could demonstrate this by having the evaluations run not just within participants with their own mental representations, but also with the mental representations of other participants, where they could find some difference in the evaluation? E.g. a participant could evaluate the reconstruction of another participant as lower than their own in terms of reflecting a given emotion?

Thanks for this comment. We considered your comment, reorganized, and revised the entire section 4.2.

We added another subject evaluation experiment into the section 4.2. The corresponding evaluation results can be found in Table 2.

2) Formating / order

I don't fully understand the figure caption formatting: Why are the captions in the main text without clear separation? How can the figure caption (or what I think is the figure caption) of figure 3 end with "Here are our observations"? That would make sense if it was part of the text, but not for a figure caption.
The caption for figure 2 is almost entirely unintelligible to me. It got clearer when finding the later line 217 - 221. The authors should also explain what they mean with dominant and complementary before relying on these terms in the figure 2 caption. What is i? What is j? What do the three rows denote? What does Omega_{i,j*} mean?

- Why are the captions in the main text without clear separation?

  The format problems have been solved.

- How can the figure caption (or what I think is the figure caption) of figure 3 end with "Here are our observations"? That would make sense if it was part of the text, but not for a figure caption.

  The format problems have been solved.

- The caption for figure 2 is almost entirely unintelligible to me. It got clearer when finding the later line 217 - 221.

  We delete the previous Fig 2, since all the information can now be found in the main text.

- The authors should also explain what they mean with dominant and complementary before relying on these terms in the figure 2 caption. What is i? What is j? What do the three rows denote? What does Omega_{i,j*} mean?

  We modified the narration of methodology and defined each math terms. The definition can be found in section 2.3.

Why is the section on "convergence efficiency" listed after the mechanical turk results? It would seem more logical to me to have them in the middle between the first reverse correlation results and the validation experiment, since the same data as in the reverse correlation results are used.

Thanks for the proposal. We followed your suggestion and placed “convergence efficiency” after the Section 3.1 “Dominant and complementary AUs computation”.

Now “Convergence efficiency” can be found in section 3.2.

Further, (but that is a subjective point and I will accept it if the authors see it differently), I feel the way the methods are noted down is somewhat confusing. Why is it necessary to first abstractly note the number of trials as m in line 197, and later specify it as 840 (line 292, in the results section -- that just seems chaotic to me). In the same way, why does the setting of the threshold (line 269) have to be separated from its description in line 237?

Since these are editable hyper-parameters, according to most papers of the community, they will be specified after the methodology. We understand the concerns from the reviewer. Maybe the subsection “experiment settings” within the Section “Results” is the cause of your confusion.

Thus, we separated “Experiment settings” from the “Result” section and now set it as an individual section after the “Method” section (see section 2.5).
Table 2 Caption: The authors should explain here what they mean by "for" and "over".

We modified these words and gave examples. (See Table 3 and Section 4.2.2)

3) Referencing results to chance level
Line 322: "significant" -- what is the definition here? Can the authors add a permutation-based approach that would show that exceeding 80% is indeed something that we would not expect from chance alone? The same holds for the conclusions in line 354: Are the personal components really personal to a level that exceeds chance? Or do we just see noise that looks different for each participant?

- Line 322:

  Since all the trials are randomly generated, each trial is independent.

  In terms of human perception, if the observer randomly selected, it was 50%. That is to say the corresponding AU carries no information content. We set these thresholds because we try to align with the SOTA prototypes (that have 2 to 5 AUs activated). (See section 2.5 “Implementation: mental representation computation” lines 308-321).

- Line 354:

  About “personal”, we add a subjective evaluation to discuss it. The chance-level baselines are 18.75% and 12.5%. (See Table 2 in Section 4.2.2)

Why were the different tresholds Tq selected? Why is 51% not enough (that's more than 50%, too)? To me it seems it might make sense to derive an empirical chance level, e.g. with permutations?

We added explanation in section 2.5 “Implementation: mental representation computation”.

4) Why was observer #2 chosen for the main figures? The main figures should include group-level results.

As in majority of the publications, we provide all the statistical results (such as Tables 1-3), and figures for explanation (such as Figs 2 and 3). Indeed, it is impossible, in terms of space, to provide all the raw results in the main text.

The result of observer #2 (now corresponding to Fig 2) is an example to illustrate the common conclusion from all the results. Thus, we don’t provide all the results such as Fig 2 and Fig 3 but provide them in the supporting information (see S1-S6 Figs).

To minimize the misunderstanding, we added the sentence “Similar observations can be found in supporting information” (See the second paragraph of Section 3.1)

5) On how many trials was each dominant / complementary AU result based (i.e. how many trials were in the corresponding subsets?)

It seems that the reviewer asked this question caused by the position of "convergence efficiency" in the manuscript, indeed in the figures of "convergence efficiency", we can find the answer: the length of each curve corresponds to the number of trials per observer.

We believe that the previous comment to change the location of the section "convergence efficiency" would eliminate the difficulty in understanding and to make the reading smoother.

6) "corresponding complementary AUs have much lower proportions than the dominant AUs" -- I see exactly the opposite in the figure. Complementary AUs reach 1.0, which is achieved by none of the dominant AUs. What on earth do the authors refer to here?

Thanks for mentioning this. I think the reviewer read the wrong charts.

We indicated that the prerequisite is the "first row of Figure 2" which refer to "the dominant AU computation".

The "corresponding complementary AUs" refer to the AUs in the chart of "the dominant AU computation" not the chart of "the complementary AUs computation".

We add "for the dominant AU computation…" and an example for emphasis. (See Section 3.1 the 3rd and the 4th paragraphs)

7) Line 337: "the correpsonding AUs are listed at the bottom of the faces" -- I can't see anything there except for some undefined blobs of turquoise. The authors should consider a higher resolution of their figures.

All the figures are regenerated with 300 dpi. Please download the images via the links provided in the manuscript for better observation.

8) Line 412: The manuscript should stand on its own. It is not enough to refer to a third source to explain the "Schulze voting method". I do not understand what the authors mean by "cyclic preference".

We add the explanation about "cyclic preference." And we add a brief explanation of "Schulze method" in supporting information. (See the last paragraph of Section 4.2.1)

9) line 430: I do not see how this validates the personalised prototypes.

A subjective experiment for validating personalized prototypes is added. (See Section 4.2 and table 2)

10) line 71: do the authors validate their claim that their model can "can cover a wide range of local facial movements"?

We clarified it in lines 154-156.

11) line 115: on what dimension are "high"- vs "low-level" attributes different? How are action units "low level"?

We realized that “high” and “low” are confusing terms. The attributes are not classified by the “high” dimension and “low” dimension. We replaced them by “global attributes” and “local attributes”. See section 1.3.1.

12) The authors should cite the work of Peterson et al, PNAS 2021

According to the email from the academic editor, we can not read the link of the reference and the title of this paper is not mentioned. We think the reviewer refers to the literature titled "Deep models of superficial face judgments".

Thanks to the reviewer for mentioning this literature. It is helpful. We add this paper in the section of limitations and future work.

Typos etc:

- line 30: "artificial intelligent" -> change either to artificially intelligent (still sounds a bit weird though) or artificial intelligence
- line 51: "mental representation" -> either "the mental representation" or "mental representations"

- line 147: "was asked" -> were asked
- line 375: "not to the extensive discussion" -> "not the extensive discussion", or "not to extensively discuss"

Thanks for listing the typos. All these typos have been corrected.

6. PLOS authors have the option to publish the peer review history of their article (what does this mean?). If published, this will include your full peer review and any attached files.

Yes, please publish the peer review history.

If you choose “no”, your identity will remain anonymous but your review may still be made public.

Do you want your identity to be public for this peer review? For information about this choice, including consent withdrawal, please see our Privacy Policy.

Reviewer #1: No

Reviewer #2: No
